# Recent Advances in Postharvest Pest Biology and Management

**DOI:** 10.3390/insects12060543

**Published:** 2021-06-11

**Authors:** George N. Mbata, Michael D. Toews

**Affiliations:** 1 Agriculture Research Station, Fort Valley State University, Fort Valley, GA 31030, USA; 2 Department of Entomology, University of Georgia, Athens, GA 30601, USA; mtoews@uga.edu

## 1. Introduction

A sizable proportion (about 8%) of the world population is facing food insecurity [1]. This proportion rises to about 30% in some low-income countries [1]. Estimated postharvest losses of global food production vary depending on the crop, country and climate, but could be up to 40% [1].

Postharvest losses can be quantitative, e.g., weight loss of the product, or qualitative, e.g., changes in appearance, taste, texture, and nutritional or economic value. Postharvest damage can also affect consumers through reduced food safety, harming their health, wellbeing, and productivity. Postharvest losses associated with food quality and safety can result in lost market opportunities and resources when international trade standards cannot be met due to the presence of disease, pests, and residues of agro-chemicals. Common factors influencing research in postharvest commodity protection are the desire to increase the storability of harvested grains, thus ensuring food availability to sectors of the population that are at risk, and a reduction in pesticide residues in produce and processed food. Recent studies have focused on understanding pest composition and dynamics in storage facilities [2], the efficacy of applications of chemical pesticides and terpenes on the regulation of pest populations, and the modification of pest behavior resulting from treatment with pesticides and growth regulators [3,4,5,6].

Research aimed at the better understanding and management of postharvest pests reported in this “Recent Advances in Postharvest Biology and Management” Special Issue are introduced in the following sections.

## 2. Population Dynamics and Trapping of Postharvest Pests in Peanuts

Perez et al. reported on population dynamics and the temporal abundance of postharvest pests on peanuts within two commercial shelling facilities located in the southeastern United States [2]. In order to institute an effective pest management program in any postharvest system, active pest monitoring, the correct identification of pests, and shifts in pest populations with temperature and time have to be understood [7]. Commercial pheromone/kairomone-baited dome traps and pheromone-baited flight traps were deployed in the peanut shelling plants. The traps were serviced weekly for a year. The most abundant insect caught in dome traps was *Lasioderma serricorne* (87–88%); *L serricorne* was not caught in flight traps during winter months, but were always caught in dome traps. Other insects caught in traps were *Tribolium cataneum*, *Typaea stercorea*, *Carpophilus* spp., *Plodia interpunctella* and *Cadra cautella*. Populations of *T. castaneum* persisted throughout the monitoring period. More insects were found in the processing areas of the plants, and increased temperature was highly correlated with increased pest populations in the plants. The study concluded that pest management efforts should be concentrated at the entrance point for in-shell peanuts into the plants [2].

## 3. Fumigation of Phosphine Resistant *Tribolium castaneum* with Carbonyl Sulfide

The easiest tools available for the management of postharvest arthropod pests are fumigants because of the convenience of their applications and fast action in disinfesting commodities. Fumigants are widely used for the treatment of bulk grains or products in quarantine because of their efficacy and low cost compared to other methods [8]. Methyl bromide (MB) was a fumigant of choice, but its use has been severely restricted under the United Nations Montreal Protocol because of its deleterious impact on the ozone layer [9]. Following the restricted use of MB, phosphine (PH_3_) became widely used in the fumigation of stored products, although resulted in the development of resistance or tolerance by several postharvest insect pests [10,11]. Other fumigants have either been adopted or are under investigation as substitutes for phosphine, including sulfuryl fluoride and carbonyl sulfide [3,12].

Lee et al. [3] compared the efficacy of carbonyl sulfide and phosphine as fumigants against PH_3_ resistant (r-strain) and domestic strains (d-strain) of *Tribolium castaneum* and *Oryzaephilus surinamensis*. Exposure of developmental stages of the test insects to a concentration of 2 mg/L of PH_3_ in a fumigation chamber for 4 h resulted in 2.9% mortality of *T. castaneum* (r-strain), 49.5% (d-strain) and 99.2% of *O. surinamensis*. However, extending the exposure period to 24 h resulted in a 100% mortality in all life stages of the beetles. Application of COS at a concentration of 40.23 mg/L and 50 g/m^3^ in a similar fumigation chamber as that used for phosphine resulted in a 100% mortality in all the life stages of the beetles tested. Differences in mortalities were not observed between the r-strain and d-strain of *T. castaneum*. Thus, COS could be a suitable substitute for phosphine in the fumigation of commodities or produce infested by phosphine-resistant strains of *T. castaneum*. More studies are needed to determine whether COS will be effective against phosphine resistant postharvest insects.

## 4. Movement and Velocity of *Rhyzopertha dominica, Cryptolestes ferrugineus* and *Sitophilus oryzae* Exposed to an Experimental Formulation of Three Pesticides

Movement and velocity of *Rhyzopertha dominica* (F.), the lesser grain borer, *Cryptolestes ferrugineus* (Stephens), the rusty grain beetle, and *Sitophilus oryzae* (L.), the rice weevil, exposed to wheat treated with various concentrations (0–100%) of an experimental formulation of deltamethrin + methoprene + piperonyl butoxide synergist were monitored using a camera-based monitoring system (Ethovision^®^). *R. dominica* movement decreased with increases in pesticide concentration and exposure time. Movement also decreased in *Cryptolestes ferrugineus* compared to *R. dominica*, but was less impacted. The rice weevil was the least susceptible to the piperonyl butoxide synergist.

## 5. Repellency and Toxicity Effects of Essential Oils and Their Components on Weevils (*Sitophilus oryzae* L. and *S. zeamais* Motschulsky)

Many efforts are focused on plant products and extracts as alternatives to chemical pesticides in the IPM of postharvest commodities.

Klys et al. reported on the efficacy of essential oil from common caraway and L-carvone, a major compound of the essential oil, in repelling and killing *Sitophilus oryzae* [6]. The highest repellency was shown for both the essential oil and L-carvone at low concentrations (0.1–0.5), while the highest mortality was observed at higher concentrations (0.5–1.0%) [6].

Langsi et al. assessed two pure compounds (α-Pinene and 3-Carene) from cypress against the maize weevil, *Sitophilus zeamais,* for contact and fumigant toxicities. Both compounds demonstrated efficacies against both adult and immature weevils. These compounds did not discolor treated stored maize and could be exploited as novel maize insecticides [4].

The results published in this Special Issue are novel and could be useful pest management tools in certain postharvest systems.
